# Computed Tomography Perfusion Imaging: A Key Initial Test for Isolated Acute Aphasia in the Emergency Department

**DOI:** 10.31083/RN37922

**Published:** 2025-09-22

**Authors:** Eduard Bargay Pizarro, Lara Núñez Santos, Ana Valero Mut, Marc Viles García, Álvaro Ortega Sánchez, Maria Magdalena Rosselló Vadell

**Affiliations:** ^1^Department of Neurology, Hospital Universitario Son Espases, 07120 Palma, Spain; ^2^Department of Radiology, Hospital del Mar, 08003 Barcelona, Spain; ^3^Department of Neurology, Hospital de Manacor, 07500 Manacor, Spain

**Keywords:** aphasia, perfusion imaging, electroencephalography, epilepsy, ischemic stroke, neuroimaging, afasia, TC-perfusión, electroencefalografía, epilepsia, ictus isquémico, neuroimagen

## Abstract

**Background:**

Computed tomography perfusion (CTP) is a widely available imaging test in the initial assessment of acute neurological symptoms. Acute isolated aphasia is a common symptom in this group of patients, in whom an etiopathogenic diagnosis can be challenging. The aim of this study was to assess the usefulness of CTP for the initial management of this syndrome, and to evaluate whether the detection of certain perfusion patterns can be valuable in the diagnostic process.

**Methods:**

CTP scans performed in our hospital between 2019 and 2022 were retrospectively analyzed. Individuals with acute isolated aphasia who attended the emergency department within this period were included. Diagnostic, demographic, clinical, neuroimaging, electroencephalography (EEG), other complementary test, and follow-up data were collected.

**Results:**

Of the 1880 CTP exams performed, 175 (9.3%) patients presented with acute isolated aphasia, 50% of whom were female, with a median age of 71.5 (Interquartile range (IQR) 61–80) years. The etiology was vascular in 91 (52%) patients, epileptic in 26 (14.9%) patients, and due to other causes in 58 (33.1%) patients. Differences in perfusion patterns were detected between the different etiologies (*p *< 0.001), particularly in cases of epileptic origin, where hyperperfusion had a high positive predictive value for status epilepticus (83%). In this series, concrete clinical conditions such as National Institutes of Health Stroke Scale (NIHSS) score at admission and discharge, altered mental status, and fever at onset of symptoms were associated with a specific etiology.

**Conclusions:**

CTP imaging is a valuable diagnostic tool for acute isolated aphasia, enabling the optimization of acute treatment in these patients, particularly in status epilepticus and stroke.

## Abstract (es)

**Introducción:**

La tomografía computarizada de perfusión (TCP) es una prueba de imagen ampliamente disponible en la evaluación inicial de síntomas neurológicos agudos. Un síntoma frecuente en este grupo de pacientes es la afasia aguda aislada que presenta un diagnóstico etiológico de alta complejidad. El objetivo de este estudio es evaluar la utilidad de la TCP en el manejo inicial de este síndrome y determinar si la detección de ciertos patrones de perfusión puede ser de utilidad en el proceso diagnóstico.

**Métodos:**

Estudio retrospectivo de las TCP realizadas en el Hospital Universitario Son Espases entre los años 2019 y 2022. Se incluyeron pacientes con afasia aguda aislada que acudieron al servicio de urgencias durante dicho periodo. Se recogió información demográfica y clínica, estudios de neuroimagen, electroencefalograma (EEG) y otras pruebas complementarias, así como el diagnóstico y seguimiento.

**Resultados:**

De los 1880 estudios de TCP realizados en el periodo abarcado, en 175 (9,3%) se describe una afasia aguda aislada, el 50% en mujeres, con una mediana de edad de 71,5 años (IQR 61–80). La etiología fue vascular en 91 pacientes (52%), epiléptica en 26 (14,9%) y otras causas en 58 (33,1%). Se detectaron diferencias en los patrones de perfusión según la etiología (*p* < 0,001), especialmente en los casos de origen epiléptico, en los cuales la hiperperfusión mostró un alto valor predictivo positivo para estado epiléptico (83%). En esta serie, algunos rasgos clínicos específicos como la puntuación National Institutes of Health Stroke Scale (NIHSS) al ingreso y al alta, la alteración de nivel de conciencia y la presencia de fiebre al inicio de los síntomas se asociaron ciertas etiologías concretas.

**Conclusiones:**

La tomografía computarizada de perfusión es una herramienta diagnóstica de utilidad en la afasia aguda aislada, permitiendo optimizar el tratamiento agudo en estos pacientes, especialmente en casos de estado epiléptico y enfermedad cerebrovascular.

## 1. Introduction 

Around 3%–10% of patients admitted to the emergency department with acute neurological symptoms present acute isolated aphasia [1, 2, 3]. Aphasia is usually associated with other deficits, particularly in stroke patients; nonetheless, approximately 8.7% do not present concomitant neurological impairments [1, 4]. Addressing this group of patients can be challenging as they represent a broad spectrum of aetiologies, some of which require emergent treatment.

The most frequent aetiology is stroke, followed by epileptic seizures and less common causes such as aura migraine attacks, delirium, functional disorders, toxic-metabolic disorders, central nervous system (CNS) infections, CNS tumours, hypertensive encephalopathy, and posterior reversible encephalopathy syndrome, among others [2, 5].

Computed tomography perfusion (CTP) is a widely available and reliable test for acute ischaemic stroke. Its use has been described in epileptic seizures (where initial electroencephalography (EEG) is not always available [3]), during which there is hyperperfusion in the ictal phase and hypoperfusion in the post-ictal phase [6, 7, 8, 9]. CTP also plays a role in the differentiation of other stroke mimics [6, 10, 11, 12].

Acute isolated aphasia presents a diagnostic challenge with a need for a rapid diagnosis. With this in mind, we conducted a study to analyse the usefulness of CTP, an accessible tool in the emergency setting, in combination with clinical data for the differential diagnosis of acute isolated aphasia.

## 2. Materials and Methods

### 2.1 Design, Subjects and Variables

A retrospective, observational, cross-sectional study, which included patients admitted to the emergency department of our hospital with acute isolated aphasia between January 2019 and December 2022.

We included patients who underwent a multimodal computed tomography (CT), including non-enhanced cranial CT (NECT), CT angiography (CTA), and CT perfusion (CTP), after a neurology evaluation.

Medical records and multimodal CT scan results were consulted.

The following data were collected: demographic variables; stroke risk factors (hypertension, diabetes, dyslipidemia, smoke, ischaemic cardiopathy, and atrial fibrillation); the National Institutes of Health Stroke Scale (NIHSS) score and modified Rankin Scale (mRS) on admission and discharge; presence of altered mental status, psychomotor agitation, or fever on admission; initial NECT, CTA, and CTP; EEG findings; and performance of lumbar puncture. We also compared the affected vascular territory on CTP with the definitive diagnosis.

### 2.2 Image Acquisition Protocol and Analysis

Brain CT was performed on General Electric Lightspeed (CT Lightspeed VCT 64 GT1700, GE Healthcare, Chicago, IL, USA), First, a NECT was performed, to rule out haemorrhagic stroke. This was followed by a CTP with 8 cm of brain coverage (Volume Helical Shuttle technique) and injection of 40 mL of IV contrast. CTP maps were processed using AWServer (General Electric). Time to peak, Cerebral Blood Flow (CBF), Mean Transit Time, and Cerebral Blood Volume (CBV) maps were generated.

Hyper- and hypoperfusion were defined as lateralized visually distinctive perfusion changes of ±5 seconds on time to peak maps, ±5 mL/s on CBF maps, and ±10 mL/100 mL on CBV maps, compared to contralateral side. Areas of perfusion abnormality were defined using the standardized binary 10-point Alberta Stroke Program Early CT Score (ASPECTS). Examples of normal, hyperperfusion and hypoperfusion studies have been included in a figure the **Supplementary Material**.

In the emergency setting, CTP maps, NECT, and CTA were qualitatively described by a general radiologist. Incongruent or inconclusive results were reviewed by a neuroradiologist for the purpose of this study.

### 2.3 Electroencephalography

20-to-30-minute studies were performed using Nicolet EEG software version 5.71 (Carefusion, model: Nicolet, 44 channels, series: 456789, Natus Neurology Incorporated, Middleton, WI, USA), conforming to the 10–20 international electrode system.

### 2.4 Statistical Analysis

In the univariant analysis, Pearson’s chi-square test was used to compare qualitative variables. ANOVA or Kruskal-Wallis tests were used for quantitative variables.

Assumptions of normality were verified using the Kolmogorov-Smirnov test and the Shapiro-Wilk test. A value of *p* < 0.05 was considered statistically significant. A logistic regression analysis was performed to determine the predictive variables of vascular and epileptic aetiologies. R software, version 4.2.3 (R Foundation for Statistical Computing, Vienna, Austria) was used for the statistical analysis.

## 3. Results

One thousand eight hundred and eighty CTP studies were reviewed, revealing 175 patients (9.3%) presenting acute isolated aphasia, who were therefore included in the study.

**Fig. 1.**
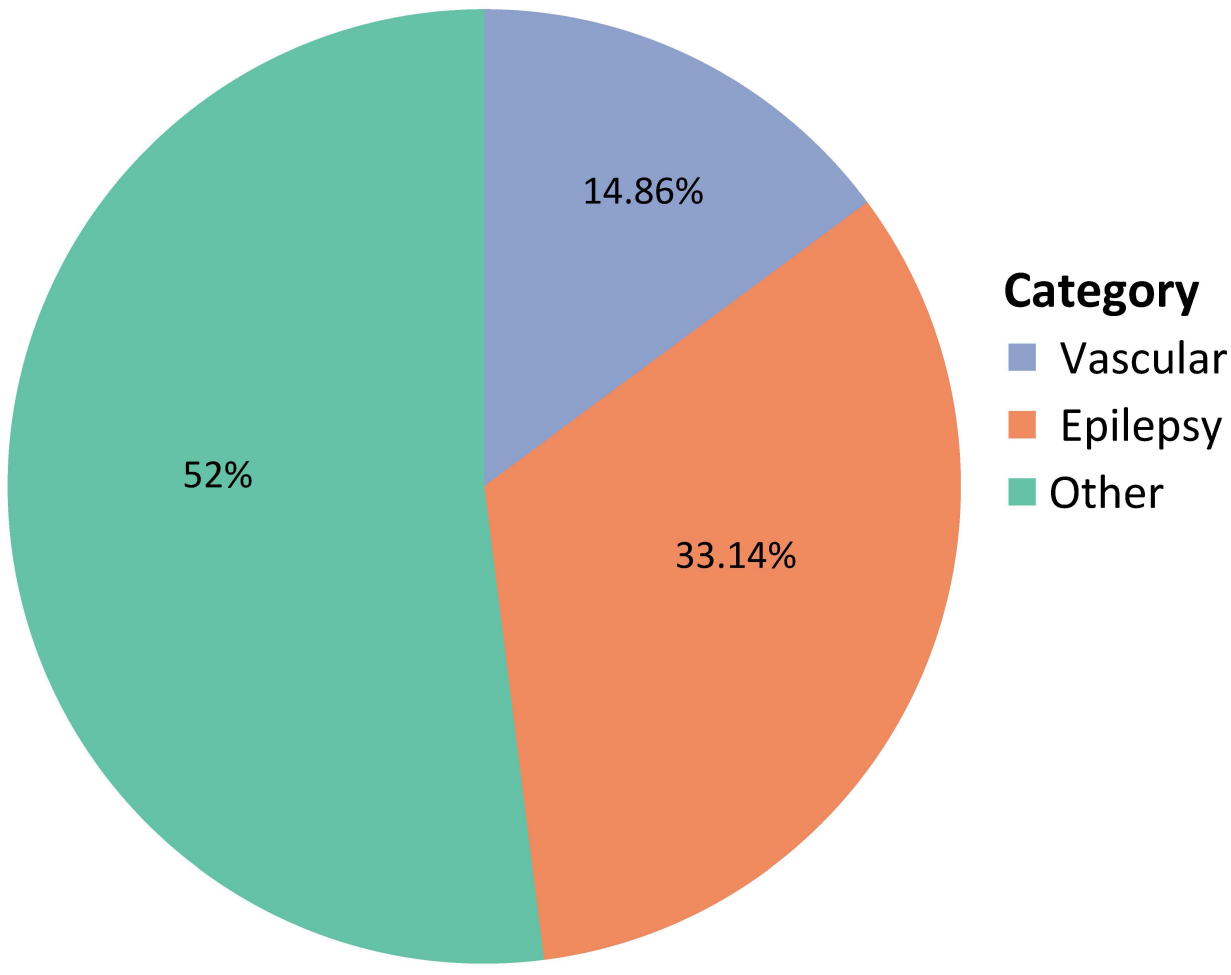
**Diagnostic categories of the included patients**. This figure represents the diagnostic categories of the patients with acute isolated aphasia: 52% were attributed to a vascular cause, 33.14% to epileptic cause, and 14.86% to other causes.

**Fig. 2.**
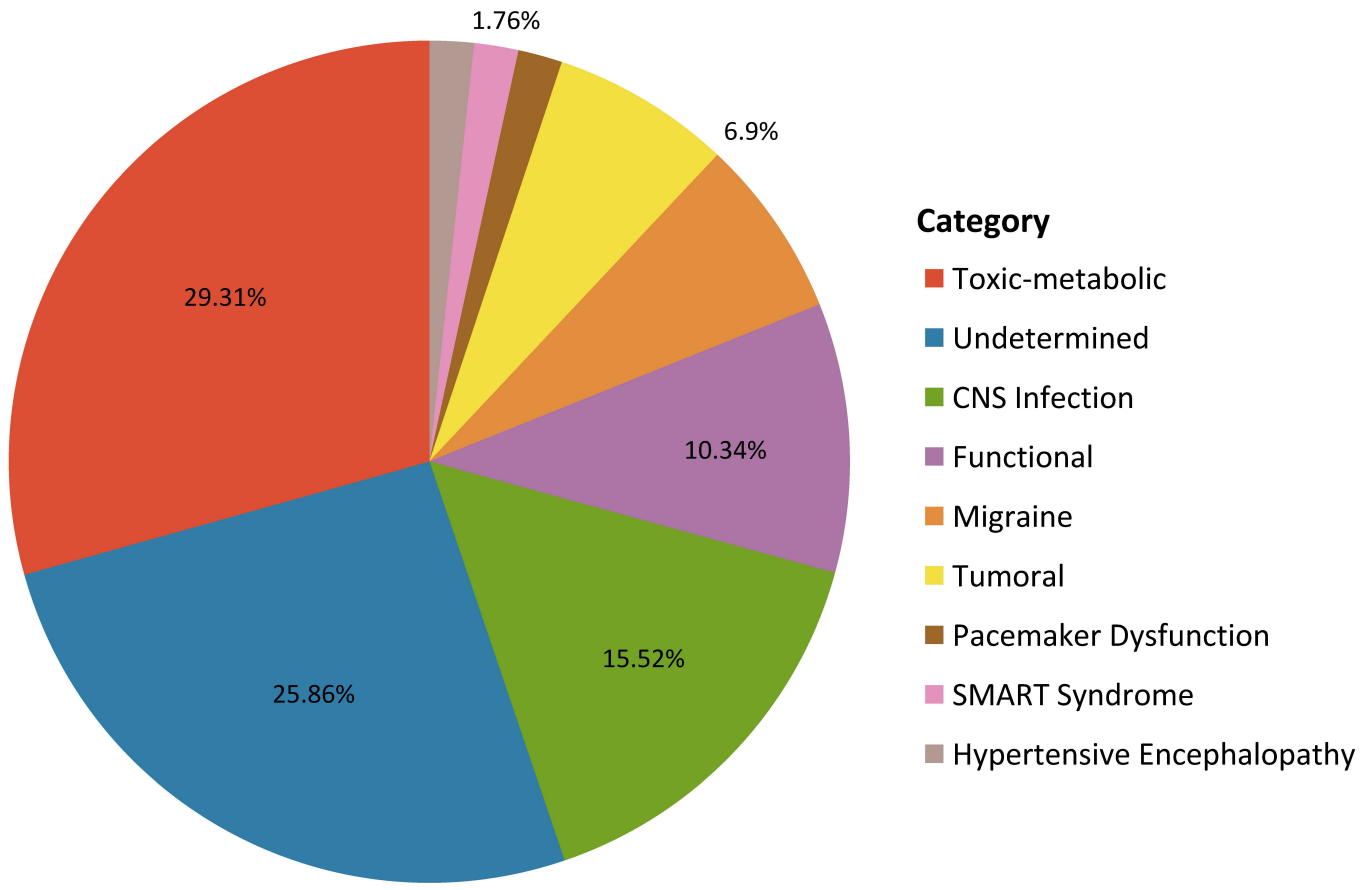
**Diagnostic subcategories of the “other” aetiologies group**. This figure represents the different diagnoses within the “other causes” category, including: toxic-metabolic (29.31%), undetermined (25.86%), central nervous system (CNS) infection (15.52%), functional (10.34%), migraine (6.9%), tumoral (6.9%), pacemaker dysfunction (1.7%), SMART syndrome (1.7%), and hypertensive encephalopathy (1.7%). SMAR, stroke-like migraine attacks after radiation therapy.

Figs. 1,2 represent the given diagnoses: vascular (52%), epileptic (14.9%), and other causes (33.1%).

Four patients had known epilepsy, while two cases were deemed epileptic, one functional and one undetermined.

Table 1 comprises the demographic variables, NIHSS score and mRS on admission and discharge, and the presence of altered consciousness, agitation, or fever on admission.

**Table 1.** **Comparisons between different aetiologies. Univariant analysis**.

Variable	Aetiology				*p* value
	Total	Vascular	Epilepsy	Other	
Age	70.0 (22.0)	70.0 (22.50)	75.0 (17.50)	69.0 (21.75)	0.294
Sex (Women)	78 (44.57%)	31.0 (34.07%)	12.0 (46.15%)	35.0 (60.34%)	0.007*
Hypertension (HTA)	101 (57.71%)	55.0 (60.44%)	15.0 (57.69%)	31.0 (53.45%)	0.701
Diabetes Mellitus (DM)	60 (34.29%)	30.0 (32.97%)	5.0 (19.23%)	25.0 (43.10%)	0.096
Dyslipidaemia (DLP)	83 (47.43%)	46.0 (50.55%)	15.0 (57.69%)	22.0 (37.93%)	0.169
Atrial Fibrillation (AF)	37 (21.14%)	23.0 (25.27%)	5.0 (19.23%)	9.0 (15.52%)	0.352
Ischaemic Cardiopathy	28 (16.0%)	17.0 (18.68%)	3.0 (11.54%)	8.0 (13.79%)	0.563
Epileptic History	4 (2.29%)	0.0 (0.00%)	2.0 (7.69%)	2.0 (3.45%)	0.053
NIHSS at Admission	5.0 (6.0)	3.0 (5.00)	8.0 (4.00)	5.0 (7.00)	<0.001*
NIHSS at Discharge	0.0 (0.0)	0.0 (1.00)	0.0 (0.00)	0.0 (0.00)	<0.001*
mRS at Admission	0.0 (0.0)	0.0 (0.00)	0.0 (0.00)	0.0 (0.00)	0.989
mRS at Discharge	0.0 (2.0)	0.0 (2.00)	0.0 (2.00)	0.0 (0.00)	0.124
Impaired Awareness	47 (26.86%)	0.0 (0.00%)	22.0 (84.62%)	25.0 (43.10%)	<0.001*
Agitation	16 (9.14%)	1.0 (1.10%)	1.0 (3.85%)	14.0 (24.14%)	<0.001*
Fever	23 (13.14%)	6.0 (6.59%)	3.0 (11.54%)	14.0 (24.14%)	0.008*

*significant *p* value. NIHSS, National Institutes of Health Stroke Scale; mRS, modified Rankin Scale.

When comparing these variables between the diagnostic groups, statistically significant differences were found in sex, NIHSS scores at admission and discharge, and the presence of altered consciousness, agitation, and fever.

After adjusting admission variables in a logistic regression model, only NIHSS at admission (*p* < 0.001), agitation (*p* = 0.023) and impaired awareness (*p* < 0.001) remained significantly associated with the final aetiology.

### 3.1 Neuroimaging

NECT and CTA scan findings are summarized in Table 2.

**Table 2.** **NECT and Angio-CT findings classified by aetiology**.

		NECT findings				Angio-CT findings		
		Structural lesion/Established infarct	Microangiopathy/Atrophy	Normal	Early signs of stroke	Normal	Stenosis	Occlusion
Diagnosis	Epilepsy	13 (50%)	7 (26.9%)	6 (23.1%)	-	26 (100%)	-	-
	Other	5 (8.62%)	18 (31.0%)	35 (60.3%)	-	57 (98.3%)	1 (1.72%)	-
	Vascular	6 (6.59%)	21 (23.1%)	44 (48.4%)	20 (22.0%)	46 (50.5%)	9 (9.89%)	36 (39.6%)

Differences were significant (*p* < 0.001). NECT, non-enhanced cranial CT; CT, computed tomography.

NECT was normal in 23% of epileptic seizure cases and 48.4% of vascular causes, where early signs of infarction were observed in 22% of patients (median Alberta Stroke Programme Early CT Score (ASPECTS) score of 10 (IQR 10–10), 20.8% below 10 points) (*p* = 0.007).

Structural brain lesions were more common in epilepsy-related aphasia (50%) compared to vascular (7%) and other causes (9%: three patients with space-occupying lesions, one with Stroke-like Migraine Attacks after Radiation Therapy (SMART) syndrome, and one of undetermined cause) (*p* < 0.001).

Microangiopathy was distributed equitably between the vascular and epileptic groups (23% and 26.9%) (*p* = 0.685).

CTA was normal in 51% of cerebrovascular cases. One patient with a toxic-metabolic encephalopathy secondary to systemic infection had a left middle cerebral artery (inferior M2) stenosis of unknown significance (inconclusive CTP due to motion artifact). No relevant alterations were observed in the CTA in any other patients.

CTP scan findings are collected in Table 3.

**Table 3.** **CT perfusion findings according to aetiology**.

		CT perfusion findings			
		Hyperperfusion	Hypoperfusion	Not evaluable	Normal
Diagnosis	Epilepsy	5 (19.2%)	2 (7.69%)	4 (15.4%)	15 (57.7%)
	Other	1 (1.72%)	3 (5.17%)	4 (6.88%)	50 (86%)
	Vascular	0 (0%)	53 (58.2%)	1 (1.10%)	37 (40.7%)

Differences were significant (*p* < 0.001).

Perfusion outcomes exhibited significant variations when compared between aetiologies (*p* < 0.001):

- Ischaemic cerebrovascular aetiology: 58% hypoperfusion, 41% normal, 1% inconclusive, 0% hyperperfusion. All CT hypoperfusion patterns were congruent with a vascular territory.

- Arterial occlusion: 97.2% had congruent hypoperfusion; the remaining case was inconclusive.
- Arterial stenosis: 55% showed congruent hypoperfusion, 45% showed no perfusion impairment.
- Normal CTA: 28% showed an area of hypoperfusion corresponding to a more distal vascular territory; 72% normal perfusion.

- Epileptic aetiology: 57.7% normal, 7.69% hypoperfusion, 19.2% hyperperfusion, and 15.4% inconclusive (motion artifact).

- In the group of other causes: 86% normal, 5.17% hypoperfusion, 1.72% hyperperfusion, and 6.88% inconclusive (motion artifact).

The perfusion patterns in epilepsy cases or other causes did not correspond to a vascular territory.

Within the 26 patients with aphasia due to epileptic aetiology, we analysed those with status epilepticus (SE) and those considered as post-ictal aphasia.

- Status epilepticus (confirmed with EEG): eight (30.8%); with 62.5% showing a pattern of hyperperfusion, 12.5% normal, and 25% inconclusive (motion artifact). The positive predictive value of hyperperfusion for diagnosing status was 83%; there was only one patient with an EEG not compatible with status epilepticus, with a final diagnosis of CNS infection with this pattern. The negative predictive value was 99%.

- Possible postictal aphasia: 18 (69.2%); with 77.8% normal CTP, 11.1% hypoperfusion, and 11.1% inconclusive.

These differences were significant (*p* < 0.001).

When comparing the other variables between patients diagnosed with status and those with postictal aphasia, statistically significant differences were observed only in the NIHSS upon arrival: seven (IQR 5–8) and nine (IQR 8.25–9) in postictal and status, respectively.

**Fig. 3.**
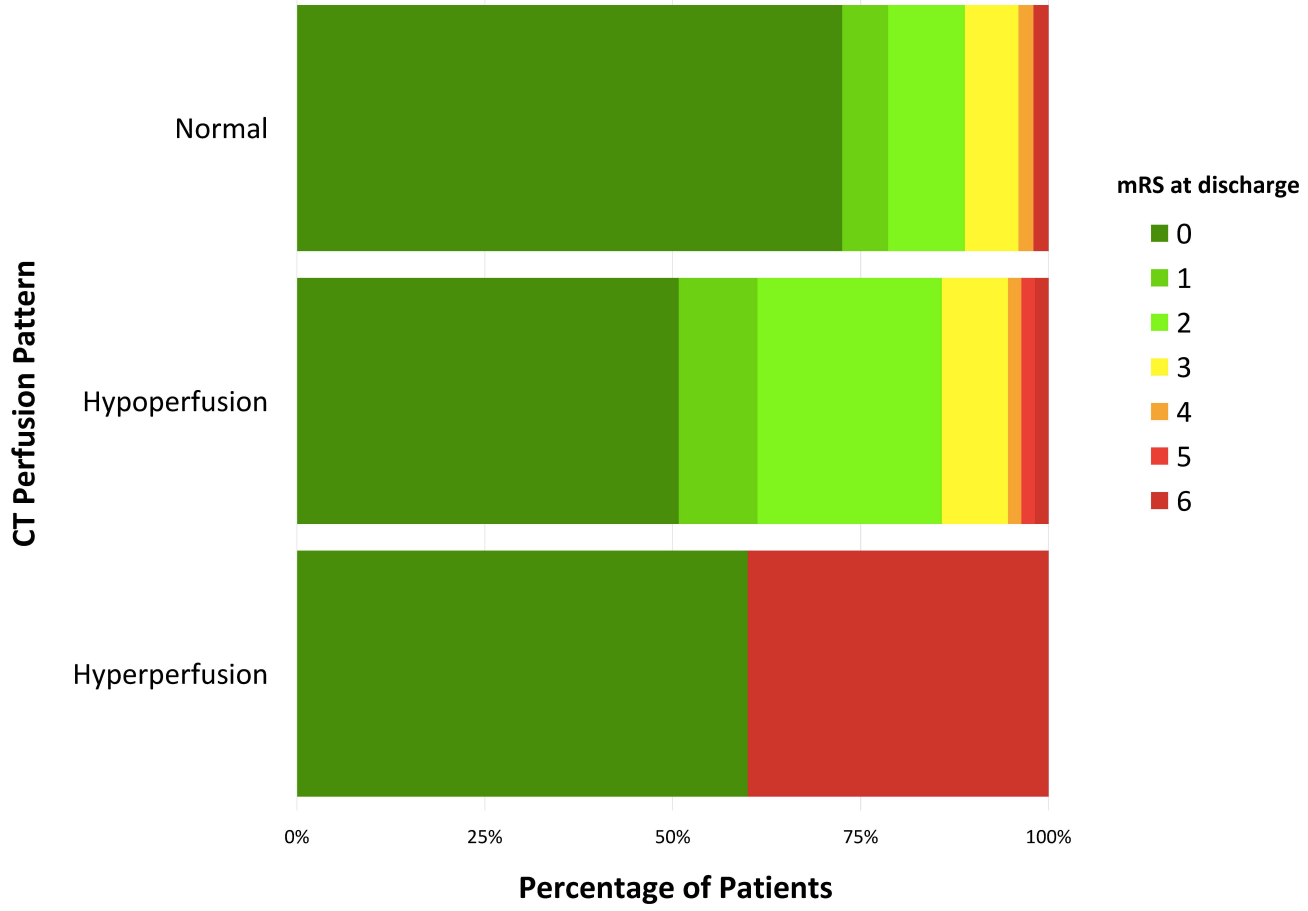
**Modified Rankin Scale (mRS) score at dicharge for each CT perfusion pattern group**. Fig. 3 represents the mRS score, from 0 to 6, at discharge for each CT perfusion group. Within the normal perfusion group (102 patients) at discharge, the distribution was the following: mRS 0, 72.4%; mRS 1, 6.1%; mRS 2, 10.2%; mRS 3, 7.1%; mRS 4, 2.0%; and mRS 6, 2.0%. In the hypoperfusion group (58 patients) at discharge, the distribution was the following: mRS 0, 50.9%; mRS 1, 10.5%; mRS 2, 24.6%; mRS 3, 8.8%; mRS 4, 1.8%; mRS 5, 1.8%; and mRS 6, 1.8%. In the hyperperfusion group (6 patients) at discharge, the distribution was the following: mRS 0, 60%; and mRS 6, 40%.

We also analysed the relationship between functional prognosis measured by mRS at discharge based on the results of the CTP, observing a worse functional outcome in patients who showed hypoperfusion in the initial CT (*p* = 0.013) with a median of 0 (0–2) vs 0 (0–1) in those with normal CTP (Fig. 3).

### 3.2 Electroencephalography and Lumbar Puncture

An EEG was performed on 78 patients (44.6%). It was carried out on all patients with epileptic aetiology, on 15 (16.5%) with vascular causes, and on 37 (63.8%) with other aetiologies: with 92.3% of epileptic patients showing abnormal EEG findings, 50% with slow activity, and 50% with spike-wave abnormalities. Seven out of eight SE showed a spike-wave pattern, whereas one of them had generalized delta-activity fulfilling Salzburg criteria.

Among the vascular aetiology group, 66.6% were normal while 33.3% showed slow activity. Regarding CNS infection, 42.8% were normal, 42.8% showed slow activity, and 14.3% showed spike-wave abnormalities. In toxic-metabolic aetiology, 30% were normal, 70% showed slow activity, and none showed spike-wave abnormalities. Both migraine patients where EEG was performed showed slow activity. In tumoral aetiology, one was normal whereas two showed slow activity.

A lumbar puncture was performed on 36 patients (20.6%): 5% vascular group, 25% epilepsy group (34.6% of all epileptic diagnoses), and 69% other group.

## 4. Discussion

In this study, we assessed the role of CTP in the diagnosis of acute isolated aphasia in 175 patients. The present study represents, to our knowledge, the longest series of acute isolated aphasia cases published to date.

### 4.1 Aetiologies

In our series, 9.3% of patients who presented acute neurologic deficits had acute isolated aphasia: the majority of vascular origin, followed by epileptic, and other aetiologies. This distribution mirrors findings from other studies, with acute isolated aphasia accounting for 3–9% of acute neurological cases, primarily vascular, followed by epileptic [1, 2, 3]. Nevertheless, in our study we found a higher prevalence of toxic-metabolic and infectious aetiologies and lower incidence of migraine auras. Several factors might explain this discrepancy: in our series, all patients were evaluated by a neurologist at the onset of symptoms; only patients who underwent CT perfusion were included, therefore, self-limiting cases were excluded.

### 4.2 Demographic Data

In our series, there was a lower percentage of women in the vascular and epileptic group. Age distribution was similar across all groups: there was a high prevalence of women in the toxic-metabolic (70%) and tumour (75%) groups.

### 4.3 Clinical Variables

In line with existing evidence [2], our series reflects the importance of clinical variables in differentiating aetiologies, the most important being NIHSS score, and presence of altered consciousness, agitation, and fever. In Polverino *et al*.’s series [2], a greater prevalence of atrial fibrillation and ischaemic heart disease was described in cerebrovascular cases. In our series, we observed this same trend, although not statistically significant, despite the evident higher stroke risk in these two pathologies. This non-significant trend was also observed in the rest of the recorded vascular risk factors (except dyslipidaemia).

A higher NIHSS score was found in patients with an epileptic cause compared to cerebrovascular, probably due to inattention.

Altered consciousness was specific to non-vascular conditions (not observed in vascular cases), appearing in epileptic causes (84%), toxic-metabolic causes (64%), and CNS infections (77%).

Agitation upon arrival at the emergency room was primarily observed in CNS infections (67%) and toxic-metabolic alterations (29%). It was anecdotal in the epileptic cause, where a single focal seizure does not usually cause an aggressive post-ictal state [13], and in vascular causes.

Fever was mainly observed in CNS infections (44%), toxic-metabolic (29%), and less frequently in epilepsy (12%).

The general functional prognosis was positive for all patients, with a median NIHSS upon discharge of 0. However, there was a significant trend towards a higher NIHSS at discharge in stroke cases, possibly explained by the residual structural damage, which was not present in the remaining aetiologies, save tumours (NIHSS 0 on discharge in all patients, possible transient aphasia in context of oedema or undiagnosed epileptic seizure).

### 4.4 CT Perfusion

Regarding the usefulness of CTP, several studies have assessed its usefulness in differentiating “stroke mimics” [6, 10, 11, 12], especially in epileptic seizures. However, few have evaluated its use specifically in cases of acute isolated aphasia [3, 4], most of which are case series or case reports [14, 15]. In our series, 58% of vascular patients showed hypoperfusion on CT, all following a vascular topographic distribution. We had no cases of hyperperfusion, although it has been described in ischaemic stroke following reperfusion [4]. In previously published series, hypoperfusion is observed more frequently than in ours [3, 4]. This could be due to variability between observers, small distal occlusions without CT-visible perfusion impairment [4, 16], and different technical characteristics between centres.

Among the cases of status epilepticus, after excluding inconclusive tests due to artifacts (25%), 83.3% showed hyperperfusion. Movement artifact due to lack of cooperation is a noteworthy limitation in this subgroup of patients, however CTP proves useful in those who do cooperate. This limitation could be methodically addressed with both pharmacological and non-pharmacological measures.

The data provided adds to existing evidence. In a study conducted by Payabvash S *et al*. [6], 77% of patients with epileptic seizures showed hyperperfusion when studied within the first three hours from onset. In a study by Jaraba Armas S *et al*. [17], which assessed patients with aphasic status epilepticus, in two patients where CTP was available, hyperperfusion was described. In the case series reported by Serven V *et al*. [14], the use of CTP is described in two cases of status epilepticus with hyperperfusion. In a study conducted by Manganotti P *et al*. [3], in which they evaluated patients with acute isolated aphasia, eight out of eleven patients with epileptic aetiology showed hyperperfusion with concordant epileptiform abnormalities on EEG. The remaining three patients showed hypoperfusion along with EEG slowing, consistent with post-ictal state [3]. In our series, most post-ictal patients presented normal perfusion while only 11.1% had hypoperfusion (including those with EEG slowing), contrasting with other studies where post-ictal state most commonly presents hypoperfusion. This could be due to differences in the time between symptom onset and the performance of the test, as well as the methodology of CTP which varies between studies. Consistently with other studies, the hypoperfusion pattern was not compatible with a vascular cause.

When analysing perfusion alterations in the other aetiologies group, hypoperfusion was described in cases of migraine, toxic-metabolic aetiology, and CNS infection; meanwhile, hyperperfusion was observed in a case of CNS infection. This perfusion pattern has been described in patients with migraine [18, 19, 20], CNS infection [20, 21], and toxic-metabolic cases in posterior reversible exncephalopathy syndrome (PRES) [20]. A key finding is the hypoperfusion pattern observed in non-vascular aetiologies, whether epileptic or not.

### 4.5 Non-enhanced Cranial CT

In our series, structural lesions or an established infarct were observed in 50% of patients with an epileptic cause. This is probably explained by the greater risk of focal epilepsy in these patients [22].

### 4.6 Prognosis

The short-term prognostic value of CTP is noteworthy. Alterations in perfusion, be they hypo- or hyperperfusion, are correlated with worse functional prognosis at discharge. One potential explanation is that it could represent the impact of the underlying cause in non-vascular aetiologies, while in vascular causes it implies reduced blood flow to the affected area.

An interesting finding in patients with hyperperfusion was that the end results were extreme: either fatal or survival without sequelae, with no middle ground. This reinforces the need for urgent and accurate decision-making in these patients.

### 4.7 Limitations

The limitations of this study include the fact that it is a single-centre study and that some CTP studies were interpreted by radiologists not specialised in neuroradiology. The latter, however, strengthens the results by showing a reality closer to actual clinical practice.

## 5. Conclusions

This research underscores the significance of a thorough patient history and physical exam in combination with diagnostic testing in making an accurate diagnosis in the setting of acute isolated aphasia.

Given the findings in the present study, coupled with the literature published to date, CTP can play a decisive role in managing patients with acute isolated aphasia, providing a faster diagnostic approach in time-dependent causes such as stroke, status epilepticus, or CNS infection. A hyperperfusion pattern is highly suggestive of status epilepticus, whereas when facing hypoperfusion, a vascular vs non-vascular pattern should be sought. The present study highlights the need for a comprehensive patient analysis grounded in robust clinical assessment along with individualized ancillary tests.

## Disclosure

The abstract was published on Spanish Society of Neurology:

Rosselló Vadell MM, Bargay Pizarro E, Viles García M, Valero Mut A. Usefulness of CT perfusion in the differential diagnosis of isolated acute aphasia in the emergency department. Neurology Perspectives. 2022; 17: 7336. Available at: https://www.elsevier.es/es-revista-neurology-perspectives-17-congresos-lxxiv-reunion-anual-sociedad-espanola-151-sesion-neuroimagen-p2-7336-comunicacion-utilidad-de-la-tc-perfusion-88847.

## Availability of Data and Materials

All data reported in this paper will also be shared by the lead contact upon request.

## Supplementary Material

Supplementary material associated with this article can be found, in the online version, at https://doi.org/10.31083/RN37922.
