# Supplementary material for: Computed Tomography Perfusion Imaging: A Key Initial Test for Isolated Acute Aphasia in the Emergency Department
Source: Rev Neurol. 2025 Sep 22;80(8):37922. doi: 10.31083/RN37922 (PMC12516825; doi:10.31083/RN37922)
Supplement: Supplementary file 1 [file 1576-6578-80-8-37922-s1.pdf]

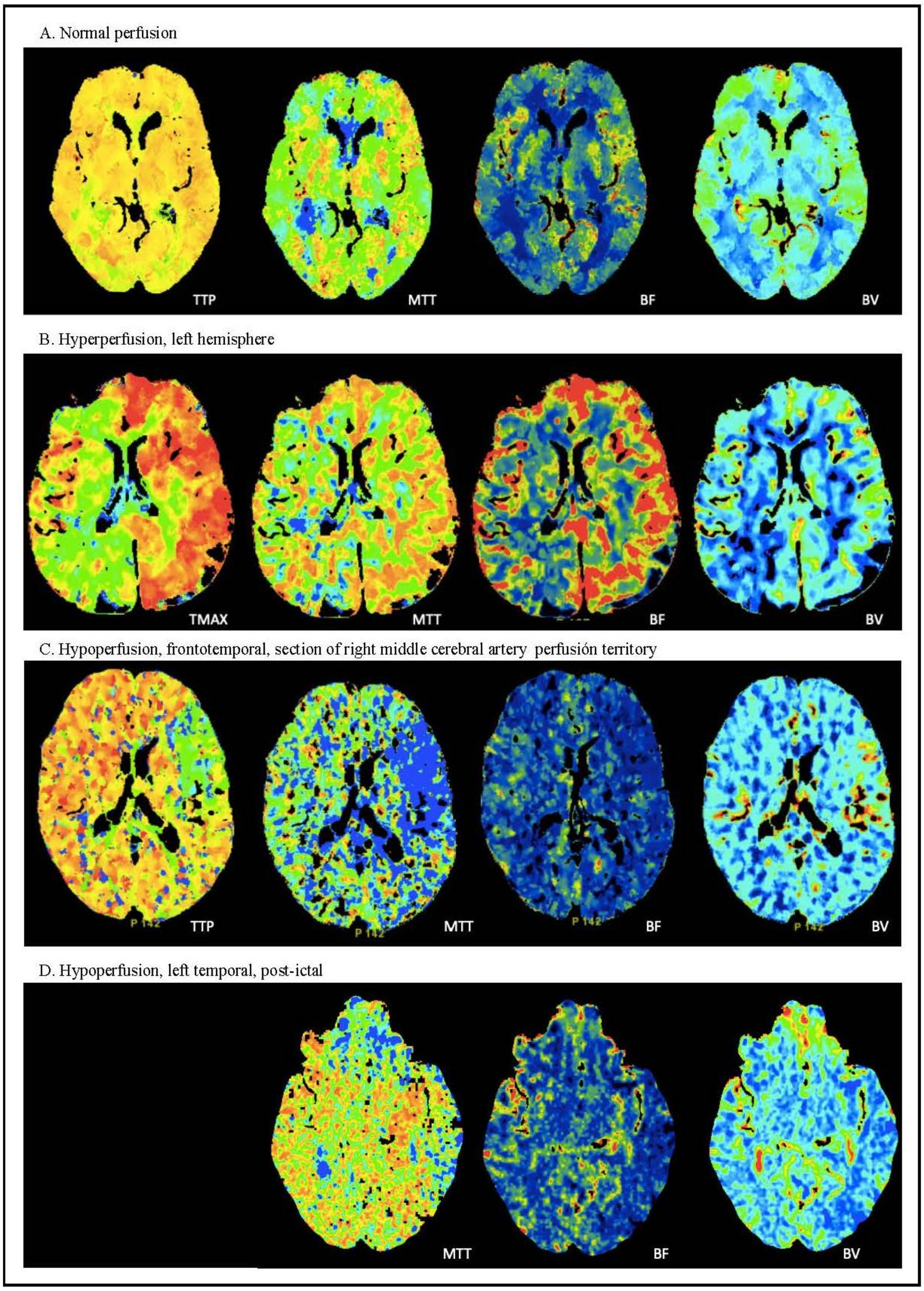

Supplementary Fig. 1. Examples of Perfusion-CT Patterns. Image A shows a normal perfusion-CT pattern. Image B represents a hypoperfusion pattern in the left hemisphere in the context of an epileptic seizure. Image C represents a hypoperfusion pattern in a patient with an acute ischemic stroke. Image D shows a hypoperfusion pattern in the left temporal region, not following a clear vascular territory in a post-ictal setting. BV: blood volume, BF: blood flow, MTT: mean transit time, TTP: time to peak, TMAX: time to maximum.
